# Characteristics of childhood cancer survivors attending a specialized survivorship clinic in the Deep South

**DOI:** 10.1007/s11764-024-01636-w

**Published:** 2024-08-07

**Authors:** Anna L. Hoppmann, Chen Dai, Lindsey Hageman, Liton Francisco, Jada Knight, Angela Mast, Kimberly Whelan, Smita Bhatia, Wendy Landier

**Affiliations:** 1 University of South Carolina School of Medicine, Columbia, SC USA; 2 Prisma Health, Columbia, SC USA; 3 Institute for Cancer Outcomes and Survivorship, University of Alabama at Birmingham, Birmingham, AL USA; 4 Division of Pediatric Hematology/Oncology, Department of Pediatrics, University of Alabama at Birmingham, Birmingham, AL USA

**Keywords:** Child, Adolescent, Cancer, Survivorship, Healthcare access

## Abstract

**Purpose:**

Childhood cancer survivors carry a high burden of late-occurring treatment-related morbidity. Long-term risk-based anticipatory surveillance allows for early detection and management of complications. We sought to examine demographic, clinical, and social characteristics associated with survivorship clinic attendance at the Taking on Life after Cancer (TLC) Clinic at the Children’s Hospital of Alabama.

**Methods:**

The cohort included 1122 TLC-eligible patients diagnosed with cancer between 2000 and 2016. The outcome of interest was ≥1 TLC visit. Univariable logistic regression modeling assessed cancer type, treatment era, age, sex, race/ethnicity, payer type, rural/urban residency, and distance from clinic. Significant variables (*P*<0.1) were retained in multivariable modeling.

**Results:**

The median age at diagnosis was 7 years old (0–19); 47% were female, 69% non-Hispanic White, 25% African American; 45% leukemia or lymphoma, 53% solid or CNS tumor, 3% other. We found that among 1122 survivors eligible to attend a survivorship clinic in the Deep South, only 52% attended. Odds of attendance were lower among survivors diagnosed at an older age, those with cancers other than leukemia/lymphoma, those lacking private insurance, and those living farther from the clinic. Race/ethnicity and rurality were not associated with clinic attendance.

**Conclusion:**

Just over half of eligible survivors attended survivorship clinic. Factors associated with non-attendance can be used to guide development of intervention strategies to ensure that childhood cancer survivors receive optimal long-term follow-up care.

**Implications for Cancer Survivors:**

Measures of healthcare access (insurance status and distance to care) were identified as potential intervention targets to improve uptake of survivorship care.

## Introduction

With ongoing advances in childhood cancer treatment, the population of childhood cancer survivors in the United States continues to grow and now includes approximately 500,000 survivors [1]. The therapies required for cancer cure place childhood cancer survivors at substantial risk of late effects including cardiac dysfunction, cerebrovascular disease, and secondary malignant neoplasm [2–4]. The significant burden of late morbidity among childhood cancer survivors continues over the life course. By the time they reach middle age, the vast majority of childhood cancer survivors will have at least one serious or life-threatening health condition [5].

The Children’s Oncology Group has developed long-term risk-based anticipatory surveillance guidelines based on prior therapeutic exposures [6]. This allows a tailored approach for childhood cancer survivors to receive the ongoing care and surveillance required across the lifespan for prevention, early detection, and timely management of complications. In this study, we determine the prevalence of eligible patients at our institution—an academic medical center in the Deep South—who are receiving the recommended survivorship care. Further, we seek to determine factors associated with receipt of survivorship care including potential intervenable barriers to survivorship care at the patient and institutional level.

## Materials and methods

The source dataset was provided by the Alabama Statewide Cancer Registry (ASCR) and includes children and adolescents ≤ 19 years of age, diagnosed with cancer at the Children’s Hospital of Alabama (CoA) between 2000 and 2016. Patients were included in the present study if they were alive and in remission at 2 years from the documented or projected end of systemic treatment (EOT). Those who relapsed or died within 2 years from EOT or had duration of follow-up less than 2 years from EOT were excluded. We included those treated with radiation alone. Those lacking systemic treatment (e.g., observation, surgery) or who had transferred medical care to another institution before reaching survivorship were also excluded.

The Taking on Life after Cancer (TLC) clinic at the Children’s Hospital of Alabama provides risk-based care to childhood cancer survivors, beginning at 2 years following the completion of therapy and has no upper age limit. When survivors meet eligibility criteria (i.e., history of systemic treatment for cancer, 2 years off therapy, in remission), the TLC Clinic coordinator telephones the survivor (or their parent/guardian), explains the survivorship program, and invites them to schedule an appointment. All eligible survivors are invited to attend the clinic. Receipt of survivorship care was defined as at least one TLC visit following eligibility.

Patient level sociodemographic characteristics (i.e., age, sex, race/ethnicity, primary payer at diagnosis) and diagnostic information (i.e., cancer type, date of diagnosis) was provided by the ASCR, while additional clinical details on treatment, end of therapy, and an updated vital status and length of follow-up were extracted from the local electronic medical record (EMR) confirmed via two patient identifiers. If missing, primary payer at diagnosis was extracted via EMR. Race was categorized as non-Hispanic White, non-Hispanic Black, Hispanic, and other races/ethnicities. Insurance was categorized as public, private, no insurance or insured, and type not specified. Cancer diagnoses were categorized in accordance with the International Classification on Childhood Cancer and further grouped for univariable and multivariable modeling as acute lymphoid leukemia, acute myeloid leukemia, lymphoma, brain and central nervous system (CNS) tumors, solid tumors, and others [7]. To provide stability to the models, acute lymphoid leukemia/lymphoma was further combined as the reference group for cancer type, since there were no differences between these cancer types in terms of receipt of survivorship care. Treatment era was split at the cohort median year of diagnosis (>2009 vs. ≤ 2009) to account for institutional efforts to engage and re-establish care for survivors in the recent era. Distance to care was calculated as the sphere distance between the county of residence at diagnosis and the treating county and further analyzed as quartiles (per 25-mile increments) and dichotomized as >100 miles or ≤ 100 miles. Rural/urban continuum codes (2013) were applied to determine a county’s status and dichotomized as metro or non-metro/rural [8].

### Statistical analysis

#### Program and coding

The SAS program (version 9.4) was utilized for statistical analysis (SAS Institute Inc.).

#### Statistical methods

The aim of this study was to determine the proportion of eligible childhood cancer survivors receiving specialized care at the survivorship clinic (TLC) and factors associated with receipt of survivorship care. A further goal of the study was to identify potential areas for intervention, including barriers to accessing care, to improve adherence to survivorship guidelines in the targeted patient population. To address these aims, descriptive statistics were examined overall and by receipt of survivorship care (those with any TLC visit vs. those with no TLC visits). We applied univariable logistic regression modeling to determine the association between patient level characteristics (cancer type, treatment era, age, sex, race/ethnicity, payer plan, rural/urban residency, and distance from clinic) and receipt of survivorship care. Significant variables from univariable analysis (*P*<0.1) were retained in multivariable modeling. Univariable associations are reported as odds ratios (OR), while all multivariable associations were reported as adjusted odds ratio (aOR) with 95% confidence intervals (95%CI).

## Results

The eligible cohort included 1122 child and adolescent cancer survivors. Sociodemographic and clinical descriptive statistics for the cohort are reported in Table 1. The median age of the cohort at diagnosis was 7 years old (range 0–19 years). Females comprised 47% of the group and males 53%. By age group at cancer diagnosis the cohort included 26% young children (0–4 years), 20% school age (5–9 years), 29% younger adolescents (10–14 years), and 25% older adolescents (15–19 years). There were 69% non-Hispanic White patients, 25% non-Hispanic Black patients, 4% Hispanic patients, and 2% other races/ethnicities. The most common cancer diagnoses were solid tumors (33%), acute lymphoblastic leukemia (26%), brain and CNS tumors (20%), and lymphoma (Hodgkin and non-Hodgkin, 14% combined). Primary payers at diagnosis included private insurance (53%), public insurance (39%), not specified (6%), or no insurance (3%). The median distance to the survivorship clinic was 62 miles (max 212 miles) and 23% of the cohort lived more than 100 miles to the clinic. Twenty-five percent lived in rural/non-metro areas.

**Table 1:** Descriptive statistics for childhood cancer survivors by TLC Clinic visit status

Characteristic	Overall (*N*=1122, 100%)	No TLC visit (*N*=534, 47.6%)	TLC visit (*N*=588, 52.4%)	*P*-value^
Age group at cancer diagnosis (*n*, %)				
0–4 y	290 (25.8%)	100 (18.7%)	190 (32.3%)	<0.0001
5–9 y	227 (20.2%)	90 (16.9%)	137 (23.3%)	
10–14 y	330 (29.4%)	162 (30.3%)	168 (28.6%)	
15–19 y	275 (24.5%)	182 (34.1%)	93 (15.8%)	
Sex (*n*, %)				
Female	526 (46.9%)	232 (43.4%)	294 (50.0%)	0.0280
Male	596 (53.1%)	302 (56.6%)	294 (50.0%)	
Race/ethnicity (*n*, %)				
Non-Hispanic White	773 (68.9%)	363 (68.0%)	410 (69.7%)	0.1043
Non-Hispanic Black	285 (25.4%)	148 (27.7%)	137 (23.3%)	
Hispanic	47 (4.2%)	18 (3.4%)	29 (4.9%)	
Other	17 (1.5%)	5 (0.9%)	12 (2.0%)	
Primary payer at diagnosis (*n*, %)				
Private	589 (52.5%)	259 (48.5%)	330 (56.1%)	<0.0001
No insurance	30 (2.7%)	19 (3.6%)	11 (1.9%)	
Public	437 (38.9%)	208 (39.0%)	229 (38.9%)	
Insurance, NOS	66 (5.9%)	48 (9.0%)	18 (3.1%)	
Cancer type (*n*, %)				
Acute lymphoid leukemia	289 (25.8%)	92 (17.2%)	197 (33.5%)	<0.0001
Acute myeloid leukemia	53 (4.7%)	24 (4.5%)	29 (4.9%)	
Lymphoma (HL and NHL)	159 (14.2%)	72 (13.5%)	87 (14.8%)	
Brain and CNS tumors	227 (20.2%)	167 (31.3%)	60 (10.2%)	
Solid tumors*	366 (32.6%)	161 (30.1%)	205 (34.9%)	
Other	28 (2.5%)	18 (3.4%)	10 (1.7%)	
Distance in miles (*n*, %)				
≤100 miles	867 (77.3%)	386 (72.3%)	481 (81.8%)	0.0001
>100 miles	255 (22.7%)	148 (27.7%)	107 (18.2%)	
Rural-Urban Continuum Codes (*n*, %)				
Metro	843 (75.1%)	404 (75.7%)	439 (74.7%)	0.7000
Non-metro/rural	279 (24.9%)	130 (24.3%)	149 (25.3%)	

^*P*-value comparing TLC visit vs. no TLC visit

*Including malignant bone tumors (39), soft tissue sarcomas (91), germ cell tumors (36), hepatic (14), retinoblastoma (19), neuroblastoma (89), renal tumors (78)

Abbreviations: *TLC* Taking on Life after Cancer, *NOS* not otherwise specified, *HL* Hodgkin lymphoma, *NHL* non-Hodgkin lymphoma, *CNS* central nervous system

**Fig. 1:**
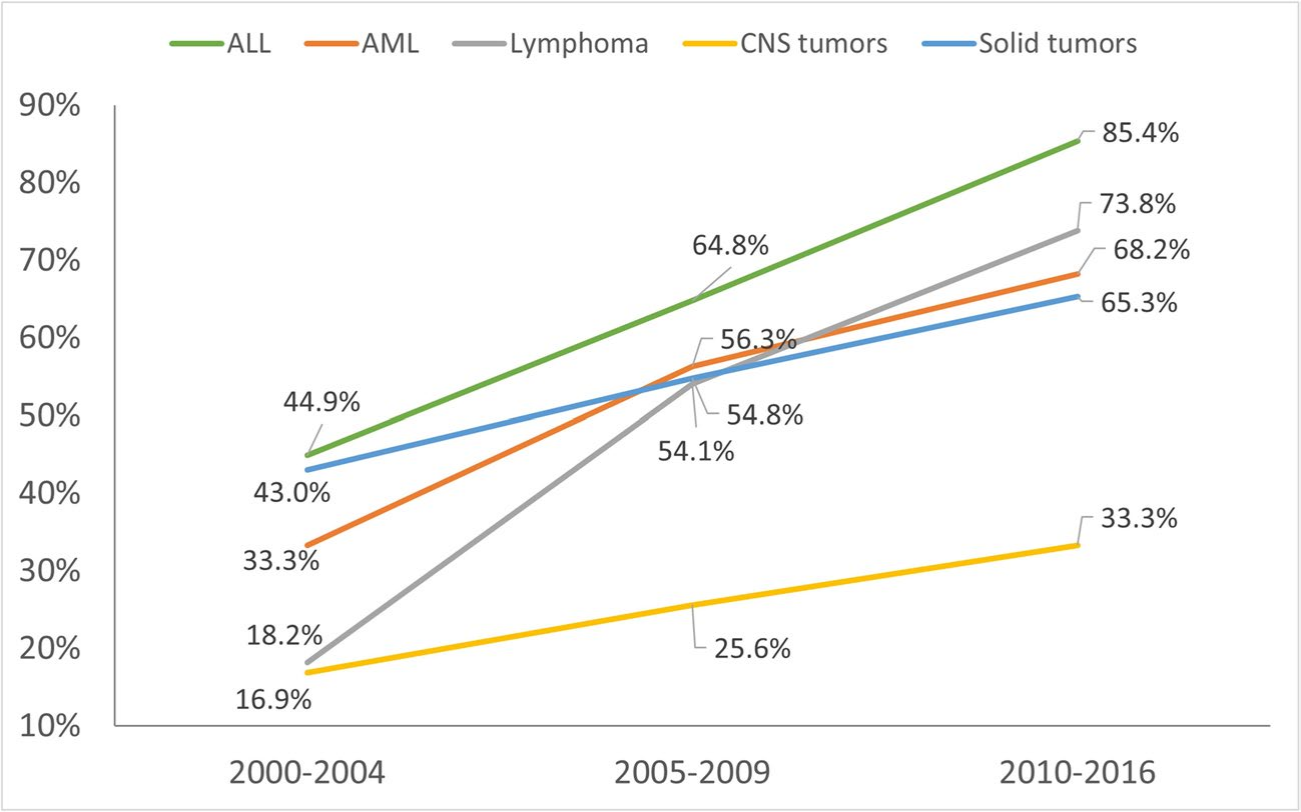
Proportion of eligible patients attending TLC by time period of diagnosis and cancer type. Abbreviations: TLC, Taking on Life after Cancer; ALL, acute lymphoid leukemia; AML, acute myeloid leukemia; CNS, central nervous system

Fifty-two percent of the cohort attended the TLC Clinic, as shown in Table 1. There were no differences in receipt of survivorship care by race/ethnicity (*P*=0.10) or rurality (*P*=0.70). A greater proportion of females (50% vs. 43% *P*=0.03), and those with private insurance (56% vs. 49%, *P*<0.0001) attended the survivorship clinic. Patients diagnosed with cancer as older adolescents (15–19 years) were least likely to attend (16% vs 34%, *P*<0.001). Those whose residence was more than 100 miles to clinic at cancer diagnosis were less likely to attend (18% vs 28%, *P*=0.0001). In terms of cancer type, there was variation in clinic attendance. Those with acute lymphoblastic leukemia were more likely to attend (34% vs 17%), while those with brain/CNS tumors were less likely to attend (10% vs 31%, *P*<0.0001). The proportion of eligible survivors attending TLC Clinic increased across cancer types with later periods of diagnosis (Fig. 1). Those diagnosed with cancer between 2010 and 2016 were most likely to attend including 33% attendance among those with brain/CNS tumors, 65% among solid tumors, 68% among those with acute myeloid leukemia, 74% among those with lymphoma, and 85% among those with acute lymphoid leukemia.

Table 2 shows univariable and multivariable associations with TLC Clinic attendance. Most variables retained their significant associations with clinic attendance in the adjusted model except for sex (*P*=0.19). Survivors diagnosed at an older age were less likely to attend the clinic (aOR=0.89, 95% CI=0.87–0.91, *P*<0.0001, per 1 year of age increase) as were those lacking private insurance (aOR=0.61, 95% CI=0.47–0.80, *P*=0.0003). Those with acute myeloid leukemia were as likely to attend the TLC Clinic as the reference group (ALL/HL/NHL, *P*= 0.28), while those with brain/CNS tumors (OR=0.17, 95% CI=0.11–0.24, *P*=<0.0001), solid tumors (OR=0.58, 95% CI=0.42–0.79. *P*=0.0006), and other rare cancers (OR=0.32, 95% CI=0.14–0.75, *P*=0.009) were less likely to attend. Those living farther from clinic at diagnosis were less likely to attend (aOR=0.90 per 25-mile increase, 95%CI 0.84–0.95, *P*=0.0007). Those diagnosed after 2009 were more likely to attend the survivorship clinic (aOR=3.05, 95%CI=2.31–4.03, *P*<0.0001).

**Table 2:** Unadjusted and adjusted logistic regression model assessing associations with TLC Clinic attendance

Variable	Unadjusted odds ratio (95%CI)	*P*-value	Adjusted odds ratio (95% CI)	*P*-value
Age at cancer diagnosis (continuous)				
Age^	0.91 (0.89–0.93)	<.0001	0.89 (0.87–0.91)	<.0001
Sex (ref: female)				
Male	0.77 (0.61–0.97)	0.0281	0.84 (0.64–1.09)	0.1894
Race/ethnicity (ref: non-Hispanic White)				
Non-Hispanic Black	0.82 (0.62–1.08)	0.1515	N/A	N/A
Hispanic	1.43 (0.78–2.61)	0.2498	N/A	N/A
Other	2.12 (0.74–6.09)	0.1608	N/A	N/A
Year of diagnosis (ref: ≤2009)				
Diagnosed after 2009	2.47 (1.93–3.16)	<.0001	3.05 (2.31–4.03)	<.0001
Primary payer at diagnosis (ref: private)				
Non-private insurance^×^	0.74 (0.58–0.93)	0.0108	0.61 (0.47–0.80)	0.0003
Cancer type (reference acute lymphoid leukemia/lymphoma)				
Acute myeloid leukemia	0.70 (0.39–1.24)	0.2192	0.71 (0.38–1.33)	0.2849
Brain and CNS tumors	0.21 (0.15–0.30)	<.0001	0.17 (0.11–0.24)	<.0001
Solid tumors	0.74 (0.55–0.97)	0.0326	0.58 (0.42–0.79)	0.0006
Other	0.32 (0.14–0.71)	0.0052	0.32 (0.14–0.75)	0.0086
Geography				
County centroid distance to treating facility (per 25-mile increase)	0.90 (0.85–0.96)	0.0005	0.90 (0.84–0.95)	0.0007
Non-metro/rural area (ref: metro)	0.95 (0.72–1.24)	0.7002	N/A	N/A

^Per 1 year increase; ^×^public insurance, no insurance, or insured NOS

Abbreviations: *TLC* Taking on Life after Cancer, *CI* confidence interval, *N/A* not applicable, *CNS* central nervous system

## Discussion

This analysis of 1122 child and adolescent cancer survivors sought to determine the prevalence of eligible survivors attending the survivorship clinic at a major academic medical center in the Deep South and to determine factors associated with receipt of survivorship care. We dichotomized the sample as those that had received at least one survivorship visit vs. those who had no visits to determine patterns and barriers in establishing survivorship care at our center. Approximately half of the eligible cohort (52%) attended the survivorship clinic. There were significant associations between clinic attendance by diagnosis era, age at diagnosis, primary payer, cancer type, and distance to the clinic. There was no difference in attendance by rurality or race. Those diagnosed after 2009 were three times more likely to attend the survivorship clinic. This reflects the significant effort at our institution to re-engage patients lost to long-term follow-up care in the most recent area; a strategy that is working, though there remain sub-groups with lower clinic attendance.

In our cohort, each additional year of age at cancer diagnosis was associated with decreased odds of survivorship clinic attendance. Further, those diagnosed as adolescents (15–19 years at diagnosis) were least likely to attend the clinic, when compared with survivors who were diagnosed at a younger age. The unique needs of adolescent and young adult (AYA) cancer survivors are well documented in the literature [9–11]. In addition to medical long-term follow-up, AYA cancer survivors have unique psychosocial needs [10]. For this reason, AYAs in particular may benefit from the resources (medical and beyond) available in a long-term follow-up clinic, and it is concerning that survivors in our cohort who were diagnosed during adolescence were least likely to attend the survivorship clinic. The reasons for lack of attendance in this older age group are likely multifactorial and may include barriers related to insurance/employment opportunities or financial challenges [12, 13]. Older childhood cancer survivors may also lack awareness about their ongoing health needs or have negative memories and beliefs about their prior cancer diagnosis and treatment [14–16].

Those with private insurance were most likely to attend the clinic, while those who were uninsured or primarily insured with Medicaid at cancer diagnosis were less likely to attend. While lacking health insurance entirely is a clear financial barrier to care and has been previously documented among childhood cancer survivors [17], the role of Medicaid coverage is more complex. The Childhood Cancer Survivor Study reported higher rates of survivorship care utilization among those with active Medicaid (in adulthood) as compared to those with private insurance [17]. In contrast, our study applied insurance status at cancer diagnosis. It is not uncommon for childhood cancer survivors to experience a change in insurance status from cancer diagnosis to survivorship, including loss of coverage. This is particularly true for survivors after reaching age of majority due to changes in eligibility for parental plans or Medicaid. Prior studies have shown that any insurance status change, particularly loss of coverage, is associated with lower utilization of cancer related follow-up care [18]. A possible explanation for lower survivorship attendance among those without private insurance in our cohort could be insurance status change or loss, highlighting the importance of screening for barriers to care including insurance enrollment across the spectrum of care (at diagnosis, during changes in treatment, the transition point into survivorship care, and when reaching the age of majority).

The published data regarding cancer type and survivorship care is inconclusive, but several studies have suggested higher attendance among those with leukemia/lymphoma and lower attendance among those with CNS tumors [19]. Our study adds to the body of literature supporting these associations [20–22]. Attendance in our cohort was highest among those with leukemia or lymphoma while those with brain/CNS tumors were least likely to attend. In our cohort, those with solid tumors or other rare cancers were also less likely to attend. Differences in receipt of survivorship care by cancer type may reflect varying system level practices and care patterns across different institutions and care contexts. Nonetheless, the association between brain/CNS tumors and low uptake of survivorship care in our practice setting warrants further exploration given the significant risk of late effects in this group.

There are conflicting data regarding rurality as a risk factor for adverse outcomes in childhood cancer. While a large national sample found no association, a smaller study in Washington State using census track level rather than county measures found rurality to be a risk factor for adverse outcomes [23, 24]. In our study, childhood cancer survivors from rural/non-metro areas at cancer diagnosis were as likely to attend the survivorship clinic as their urban counterparts. This mirrors our prior study that showed no survival differences by rurality among children with cancer in Alabama [25].

Our prior study in Alabama showed a significant association with distance to care and inferior survival among children with cancer in the state. Alabama is unique in the distribution of pediatric cancer care, with most children in the state traveling to our major academic medical center, and >20% traveling more than 100 miles to care [25]. We previously showed those living farther from care had an increased risk of death at 1 year from cancer diagnosis, and the increased risk persisted into survivorship (5 years and 10 years from diagnosis) [25]. In the present study, distance (per 25-mile increase) is associated with reduced attendance at the survivorship clinic, consistent with two previously published cohorts [22, 26]. Considering the present and past Alabama studies together illustrates that those living far from survivorship care in the state are less likely to receive long-term follow-up care in the TLC Clinic, and more likely to experience late mortality.

Lastly, race/ethnicity was not associated with survivorship clinic attendance in our cohort. Non-Hispanic Black patients were well represented in the sample and were as likely to attend the TLC Clinic as non-Hispanic White patients. This finding contrasts with prior studies that report lower odds of survivorship care among Hispanic, non-Hispanic Black, and other races/ethnicities as compared to non-Hispanic White childhood cancer survivors [14, 27–29]. The present study describes initial establishment of survivorship care, and we should continue to evaluate potential differences by race/ethnicity in longitudinal survivorship follow-up at our institution.

Our study has some limitations. A projected end of therapy date by cancer type was applied for those lacking a precisely documented end of therapy date (*N*=138). However, we tailored the estimated treatment durations to the various cancer types and combined this information with disease status and vital status to determine eligibility for survivorship care. We excluded patients who did not receive systemic therapy (e.g., surgery alone). There were also some patients for whom we did not have complete treatment records available. Additionally, the variables “primary payer” and “distance to care” were collected at the time of cancer diagnosis and may have changed by the time the child was eligible for survivorship care. This study did not assess survivors pursuing survivorship care in a primary care or other long-term follow-up setting rather than the TLC Clinic and did not evaluate longitudinal follow-up in the TLC Clinic over time. Lastly, we lacked individual level social determinants of health variables (transportation, socioeconomic status) that may impact access to care in this population.

These limitations notwithstanding, the study has several strengths including the large and diverse cohort of child and adolescent cancer survivors. The cohort reflects a large proportion of patients with residence in rural areas (25%) and far from the clinic (23% were >100 miles) allowing for analysis of geographic variables. The practice environment is also a strength as the survivorship care for our academic medical center is centralized at the TLC Clinic across ages (no upper age limit). Because attrition is a consideration regarding engagement in survivorship-focused care, the TLC Clinic targets recruitment beginning 2 years following completion of therapy, which aligns with recommendations from the Children’s Oncology Group [6]. Lastly, using the ASCR dataset as the source dataset is a strength, as the data requirements for the state registry are rigorously standardized and maintained.

In conclusion, though the reasons for not attending the survivorship clinic at our major academic medical center are diverse, they present an opportunity to intervene towards the goal of having all eligible patients establish long-term follow-up care. Moving forward, it will be important to assess longitudinal follow-up in addition to establishing survivorship care. We will study local referral practices by cancer type to understand and target potential health system improvements. We identified health care access as a key barrier towards survivorship clinic attendance, including insurance status and distance to the clinic. Insurance status is a potential intervention target that may be addressed by screening/counseling at the time of transition into survivorship and at age of majority. Barriers related to distance present the opportunity to partner with primary care physicians across Alabama and/or to leverage telehealth services to decentralize survivorship care for appropriate subgroups. In all efforts, special attention to AYA survivors is warranted, as they were least likely in our cohort to receive survivorship care.

## Data Availability

Data requests can be sent to the corresponding author. The data include protected health information on a vulnerable population, and a data use agreement would be required.

## References

[1] Surveillance, Epidemiology, and End Results, Program, SEER Cancer Statistics Review 1975-2018. Updated April 15, 2021. Accessed March 27, 2024]; Available from: https://seer.cancer.gov/csr/1975_2018/.

[2] Hudson MM, et al. Clinical ascertainment of health outcomes among adults treated for childhood cancer. JAMA. 2013;309(22):2371–81. 23757085 10.1001/jama.2013.6296 PMC3771083

[3] Dixon SB, et al. Specific causes of excess late mortality and association with modifiable risk factors among survivors of childhood cancer: a report from the Childhood Cancer Survivor Study cohort. Lancet. 2023;401(10386):1447–57. 37030315 10.1016/S0140-6736(22)02471-0 PMC10149583

[4] Landier W, et al. Surveillance for late effects in childhood cancer survivors. J Clin Oncol. 2018;36(21):2216–22. 29874139 10.1200/JCO.2017.77.0180 PMC6804892

[5] Robison LL, Hudson MM. Survivors of childhood and adolescent cancer: life-long risks and responsibilities. Nat Rev Cancer. 2014;14(1):61–70. 24304873 10.1038/nrc3634 PMC6425479

[6] Children’s Oncology Group. Long-term follow-up guidelines for survivors of childhood, adolescent and young adult cancers, Version 6.0. Monrovia, CA; 2023. 10.1001/jamaoncol.2024.6812 PMC12188901 39976936

[7] Swerdlow SH, Campo E, Harris NL, et al. WHO Classification of tumours of haematopoietic and lymphoid tissues. Lyon, France: IARC Press; 2008.

[8] USDA Economic Research Service, Rural-Urban Continuum Codes. 2013 Rural-Urban Continuum Codes; Available from: https://www.ers.usda.gov/data-products/rural-urban-continuum-codes/.

[9] Osborn M, et al. Models of care for adolescent and young adult cancer programs. Pediatr Blood Cancer. 2019;66(12):e27991. 31524328 10.1002/pbc.27991

[10] Wolfson JA, et al. Understanding causes of inferior outcomes in adolescents and young adults with cancer. J Natl Compr Canc Netw. 2023;21(8):881–8. 37549915 10.6004/jnccn.2023.7056

[11] Nathan PC, et al. Critical issues in transition and survivorship for adolescents and young adults with cancers. Cancer. 2011;117(10 Suppl):2335–41. 21523755 10.1002/cncr.26042

[12] White PH. Access to health care: health insurance considerations for young adults with special health care needs/disabilities. Pediatrics. 2002;110(6 Pt 2):1328–35. 12456953

[13] Freyer DR. Transition of care for young adult survivors of childhood and adolescent cancer: rationale and approaches. J Clin Oncol. 2010;28(32):4810–8. 20351333 10.1200/JCO.2009.23.4278 PMC3018346

[14] Nathan PC, et al. Medical care in long-term survivors of childhood cancer: a report from the childhood cancer survivor study. J Clin Oncol. 2008;26(27):4401–9. 18802152 10.1200/JCO.2008.16.9607 PMC2653112

[15] Mertens AC, et al. Improving health care for adult survivors of childhood cancer: recommendations from a Delphi panel of health policy experts. Health Policy. 2004;69(2):169–78. 15212864 10.1016/j.healthpol.2003.12.008

[16] Zebrack BJ, et al. Health care for childhood cancer survivors: insights and perspectives from a Delphi panel of young adult survivors of childhood cancer. Cancer. 2004;100(4):843–50. 14770443 10.1002/cncr.20033

[17] Casillas J, et al. Impact of insurance type on survivor-focused and general preventive health care utilization in adult survivors of childhood cancer: the Childhood Cancer Survivor Study (CCSS). Cancer. 2011;117(9):1966–75. 21509774 10.1002/cncr.25688 PMC3433164

[18] Mobley EM, et al. Insurance coverage change and survivorship care among young adult survivors of childhood cancer. Health Serv Res. 2022;57(1):159–71. 34378205 10.1111/1475-6773.13868 PMC8763279

[19] Mobley EM, et al. Disparities in pediatric cancer survivorship care: a systematic review. Cancer Med. 2023;12(17):18281–305. 37551113 10.1002/cam4.6426 PMC10524017

[20] Zheng DJ, et al. Patterns and predictors of survivorship clinic attendance in a population-based sample of pediatric and young adult childhood cancer survivors. J Cancer Surviv. 2016;10(3):505–13. 26572903 10.1007/s11764-015-0493-4

[21] Barakat LP, et al. Factors that contribute to post-treatment follow-up care for survivors of childhood cancer. J Cancer Surviv. 2012;6(2):155–62. 22170442 10.1007/s11764-011-0206-6

[22] Nathan PC, et al. Predictors of attendance at specialized survivor clinics in a population-based cohort of adult survivors of childhood cancer. J Cancer Surviv. 2016;10(4):611–8. 26868681 10.1007/s11764-016-0522-y

[23] Delavar A, Feng Q, Johnson KJ. Rural/urban residence and childhood and adolescent cancer survival in the United States. Cancer. 2019;125(2):261–8. 30311635 10.1002/cncr.31704

[24] Ohlsen TJD, et al. Population-based impact of rurality and neighborhood-level socioeconomic disadvantage on pediatric cancer mortality in Washington state. Cancer Epidemiol Biomarkers Prev. 2023;32(1):141–8. 36343539 10.1158/1055-9965.EPI-22-0897 PMC9839485

[25] Hoppmann AL, et al. Persistent child poverty and mortality in a cohort of children with cancer in Alabama. Cancer Epidemiol Biomarkers Prev. 2023;32(3):380–6. 36129811 10.1158/1055-9965.EPI-22-0353 PMC9991934

[26] Daly A, et al. Survivor clinic attendance among pediatric- and adolescent-aged survivors of childhood cancer. J Cancer Surviv. 2019;13(1):56–65. 30560348 10.1007/s11764-018-0727-3

[27] Milam JE, et al. Cancer-related follow-up care among Hispanic and non-Hispanic childhood cancer survivors: the Project Forward study. Cancer. 2015;121(4):605–13. 25345867 10.1002/cncr.29105 PMC4319982

[28] Milam J, et al. Project forward: a population-based cohort among young adult survivors of childhood cancers. JNCI Cancer Spectr. 2021;5(5) 10.1093/jncics/pkab068 PMC8462512 34585063

[29] Oeffinger KC, et al. Health care of young adult survivors of childhood cancer: a report from the Childhood Cancer Survivor Study. Ann Fam Med. 2004;2(1):61–70. 15053285 10.1370/afm.26 PMC1466633

